# The Ability of Analysts' Recommendations to Predict Optimistic and Pessimistic Forecasts

**DOI:** 10.1371/journal.pone.0073853

**Published:** 2013-10-17

**Authors:** Vahid Biglari, Ervina Binti Alfan, Rubi Binti Ahmad, Najmeh Hajian

**Affiliations:** 1 Department of Accounting, Faculty of Business and Accountancy, University of Malaya, Kuala Lumpur, Malaysia; 2 Department of Accounting, School of Management and Economics, Tarbiat Modares University, Tehran, Iran; Cinvestav-Merida, Mexico

## Abstract

Previous researches show that *buy* (growth) companies conduct income increasing earnings management in order to meet forecasts and generate positive forecast Errors (FEs). This behavior however, is not inherent in *sell* (non-growth) companies. Using the aforementioned background, this research hypothesizes that since sell companies are pressured to avoid income increasing earnings management, they are capable, and in fact more inclined, to pursue income decreasing Forecast Management (FM) with the purpose of generating positive FEs. Using a sample of 6553 firm-years of companies that are listed in the NYSE between the years 2005–2010, the study determines that sell companies conduct income decreasing FM to generate positive FEs. However, the frequency of positive FEs of sell companies does not exceed that of buy companies. Using the efficiency perspective, the study suggests that even though buy and sell companies have immense motivation in avoiding negative FEs, they exploit different but efficient strategies, respectively, in order to meet forecasts. Furthermore, the findings illuminated the complexities behind informative and opportunistic forecasts that falls under the efficiency versus opportunistic theories in literature.

## Introduction

Dutta and Gigler [2] suggest that companies have strong incentives to avoid negative Forecast Errors (FEs) or/and generate positive FEs. They propose a contractual model where the management utility is mainly based on whether the reported earnings meet or miss the forecasts. Their theoretical model assumes that both earnings forecasts and earnings management generate positive FEs. It explains and integrates both pessimistic (opportunistic) and optimistic (efficiency) forecasts behavior of companies by illuminating the effect of earnings forecasts on the earnings management.

Abarbanell and Leahvy [1] indicated that the companies' ability to manipulate earning influences the extent of earnings management. They argue that the companies with higher growth rates are more capable in manipulating profits. Abarbanell and Leahvy [1] assume that the companies that are recommended by analysts to be bought (hereafter *buy* companies) are classified as growth type companies that will enjoy high profitability. They show that these companies conduct income increasing earnings management in order to meet forecasts and generate positive FEs.

However Abarbanell and Leahvy [1] discovered that the companies recommended by analysts to be sold (hereafter *sell* companies) are unable to conduct earnings management. Among the reasons for this are that firstly, the sell companies' stock prices are less susceptible to earnings news, which may render their earnings management ineffective with regards to influencing investors' decisions. In other words, sell companies cannot effectively manipulate and increase their low profit to boost their stock prices. Secondly, sell companies possess insufficient sums of available accounting reserves and pre-managed earnings for them to achieve any relevant earning target.

According to Dutta and Gigler [2], sell companies might also suffer from communication restrictions. This seems most logical, as the lack of resources will render sell companies unable to communicate the full scope of their rich information set to investors via the manipulation of reported earnings. Therefore, communication restrictions are binding upon sell companies.

It seems that since sell companies eschew income increasing earnings management, they are both capable and more inclined to pursue income decreasing Forecast Management (FM) to generate positive forecasts errors. Thus, the aim of this research is to examine the effects of the analysts' recommendations representing the buy and sell companies on the managers' decisions towards FM.

This research enriches the literature by examining whether sell (non-growth) companies engage in negative FM to realize positive FEs. The importance of the research is that it shows whether analysts' recommendations in terms of buying or selling of the stocks have informational value that can be used by individual investors to assess the optimism or pessimism of management forecasts. Additionally, the findings obtained here would be useful for future theoretical developments.

The rest of this article is organized as follows. Section 2 reviews the literature and highlights the problem. Section 3 develops the hypothesis, while the research methodology is explained in section 4. Section 5 describes the findings, and section 6 presents the discussion and links the findings to the literature. Finally, section 7 concludes the article with a few issues on the implications of policy.

## Literature Review

According to Hirst et al. [3], optimism and pessimism of the forecasts are characteristics over which managers are most in control. However, they appear to be the least well-understood components of earnings forecasts, both in terms of theory and empirical research. There are different point of views in the literature on optimism and pessimism of the forecasts. The two dominant views on optimism and pessimism of the forecasts are explained in the following paragraphs.

The first view fits the Watts and Zimmerman [4] opportunistic perspective, and is consistent with criticisms on aligning the management's interest with an increase in stock prices, which is advocated by Jensen and Meckling [5]. The theories of FM associated with this view have primarily modeled the forecasts as an opportunity that the management will use to pre-empt litigation concerns, influence their reputation, and produce positive FEs while simultaneously influencing stock prices.

Das et al. [6] stated that since stock prices is susceptible to management's forecasts, the management tend to report a higher forecast. On the other hand, stock prices are highly susceptible to the management's FE [7], [8]. Thus, the more negative the FE is, the more it is perceived as a sign of bad news, and such bad news will most definitely lead to a dramatic fall of stock prices [9], [10]. In order to prevent such incidents, the management is inclined to engage in practices called income decreasing FM (or reporting pessimistic forecast) in order to beat forecasts and create positive earnings surprises [11]–[13].

The second view corresponds to the efficiency perspective. Deegan and Unerman [14] stated that a great deal of positive accounting researches adopted the efficiency perspective. This perspective proposes that managers will choose to use a particular accounting method, as it will most efficiently provide a record of how the organization actually performs. The management will also use forecasts to pass insider information to outsiders. In fact, by forecasting earnings, information asymmetry is reduced, leading to a reduction in the firm's cost of capital [15].

When the company's financial position is satisfactory, and the company possesses growth capability (buy companies), the management's inclination to convey positive (true) information to shareholders will increase, which will increase the management's predictions' optimism [5], [16].

Consistent with Dutta and Gigler's [2] model, for buy companies, the forecasts convey the management's true expectation to the market, which is followed by income increasing earnings management. However, for the sell companies, the forecasts do not convey true (or optimistic) information to the market, but it is used to dampen the market expectations so that the management can benefit from a positive stock price shock, which is the result of positive earnings surprise.

This research tries to highlight the factor relating to the companies' growth status that influences the management's decision to report pessimistic forecast to produce positive FEs when companies' shares are recommended to sell, and generate optimistic forecast when the companies' shares are recommended to buy. More specifically, this research tries to determine the ability of analysts' recommendations (in terms of buy or sell recommendations) in explaining the reason behind FM.

## Hypothesis Development

This study aims to examine the effects of the analysts' recommendations as buy or sell recommendations, representing the growth and non-growth companies on the managers' decisions towards forecasts management. In order to achieve this aim, four hypotheses have been developed. This section briefly explains the theoretical framework that leads to the hypotheses.

### Analysts' recommendations and pessimistic forecasts (H1)

Dutta and Gigler [2] propose an optimal communication contract where managers who reports high forecasts of income are penalized when such a report is followed by low incomes. The managers who report low forecasts however, are shielded from the risk associated with the reported earnings. They claim that some managers issue high forecasts and subsequently manipulate earnings to realize those forecasts.

Abarbanell and Leahvy [1] conducted an empirical investigation. Depending on whether analysts issue strong sell, sell, buy, and strong buy recommendations, the companies' stocks are either classified as sell or buy, where *buy* companies are assumed to be more profitable than *sell* companies.

#### Buy companies

Abarbanell and Leahvy [1] examined the buy companies and found that firstly, the stock prices of buy companies are susceptible to earnings' news. Secondly, buy companies can effectively conduct income increasing earnings management. Thus, they show that buy companies issue high forecasts and in order to avoid market punishment, they conduct income increasing earnings management to realize those forecasts.

This income increasing earnings management in buy companies is consistent with Dutta and Gigler [2] proposition, which shows earnings' management being observed only following a high forecast.

#### Sell companies

However, sell companies pursue a different strategy. Sell companies are considered low profit companies, rendering them unable to effectively conduct income increasing earnings management [1]. This assumption is due to the following reasons; firstly, since sell companies are less vigilantly monitored by investors, their stock price are less susceptible to earnings news [17], making their earnings management ineffective in influencing investors' opinions [1]. In other words, sell companies cannot effectively manipulate and increase low profit to increase stock prices. Secondly, sell companies are companies that have a meager sum of available accounting reserves and pre-managed earnings to realize any relevant earnings target [1].

Taking into account the aforementioned issues, it seems that unlike buy companies, if sell companies issue high forecasts, they cannot conduct effective earnings management to realize the forecasts afterward, and it is more than likely that they miss the forecasts. Therefore, to prevent this from happening, sell companies prefer to issue low forecasts. Therefore, the first hypothesis would be:

*H1: Sell companies issue more pessimistic forecasts than Buy companies.*

### Analysts' Recommendations and Frequency of Positive Forecast Errors (H2)

Prior researchers have confirmed the fact that since negative FE could cause a negative shock in the stock market and deteriorate management (company) status, the management engage in FM to avoid negative FEs [13], [18].

Buy companies are growth companies and enjoy high profits. Missing the forecasts in the buy companies will inevitably lead to a decrease in the stock's price. However, sell companies usually suffer from poor earnings performance, which would be a glaring evidence of managerial incompetence [1]. Missing the forecasts for sell companies would cost managers the support of stockholders, and potentially, their very own jobs [19], [20].

Since failure in realizing forecasts exposes the sell companies to severe risks, namely, litigation risk, contract termination risk and takeover risk [19], [21], [22], sell companies are expected to meet their forecasts and avoid the negative FEs more than their counterparts. Thus, it is expected that sell companies have higher frequency of positive FEs than buy companies, therefore, the second hypothesis would be:

*H2: Sell companies have higher frequency of positive forecast errors than buy companies.*

### Forecast Management and Meeting the Forecasts (H3 and H4)

Brown and Caylor [11] stated that investors unambiguously reward firms for reporting earnings that meet their forecasts and penalize firms for reporting earnings that misses their forecasts.

The companies that analysts recommend to sell (sell companies) are the companies that does not have high growth capabilities and suffer from poor performance, which would be a glaring evidence of managerial incompetence [1]. These companies are already affected by the unsatisfactory conditions of the stock market, and if they miss forecasts, they risk further deterioration of the market state. However, unlike the buy companies, the sell companies do not possess enough resources, and have less accounting flexibility to manipulate the profit and meet their respective forecasts. Hence, sell companies seek an alternative method to meet the forecasts.

Therefore, if the company is in the sell position, the management may issue lower forecasts in order to dampen the expectation of outsiders [23]. Based on the result of the firm's ordinary operations, the management would then report an earning that is equal to or higher than the forecast (report positive forecast error), as doing so will raise the bids for the company's stocks, and subsequently, increase the company's stock price.

This provides enough incentives for sell companies to decrease their forecasts in order to create future positive FEs.

Thus, if FM in sell firms is effectively conducted to realize positive FE, then companies that meet forecasts should have conducted higher income decreasing FMs than the companies that miss forecasts. Following this assumption, the following hypotheses for sell companies should be supported.

*H3: In sell companies, companies that meet forecasts have done more income decreasing FM than companies that do not meet forecasts.*

For buy companies, it is important to meet the forecasts, as negative forecast errors cause negative shocks in the stock price. Buy companies have high profitability, and therefore have enough resources to manipulate earnings [1], [2], and can efficiently manage earnings to meet their forecasts.

Thus, in the buy companies, companies that meet forecasts do not necessarily conduct income decreasing FM to meet forecasts. Thus, we expect the fourth hypothesis for the buy companies to be supported.

*H4: There is not significant difference in income decreasing FM between buy companies that meet forecasts and those that do not meet forecasts.*

## Materials and Methods

### Models

#### The Relationship of Analysts' Recommendations and Forecast Management (H1)

The first hypothesis will be tested by running the regression of FM on the Analysts' Recommendations (AR), including moderator variables (Learning effect and Difficulty) and several control variables, and the reason for the usage and measurement process of will be explained in section 4.4.
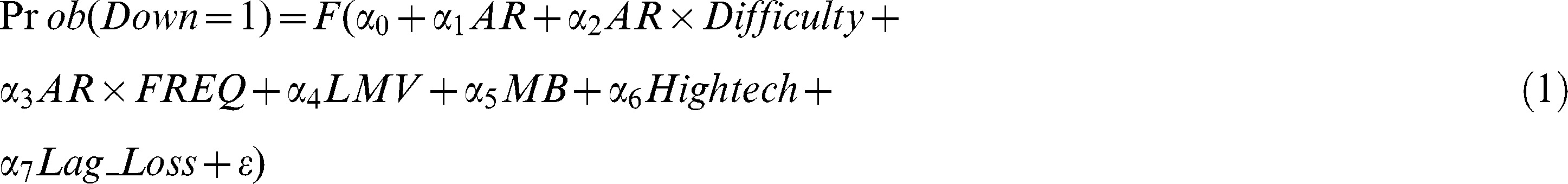
Where,

*Down = *1 if company does income decreasing FM (FM is negative) and *Down = 0* otherwise

*AR* = the Analysts' recommendations that takes the value of 1 to 5 (Section 4.2.1)

*Difficulty* = Difficulty to assess the credibility of management's forecasts

*FREQ* = frequency of FM in the previous four years as index of learning effect

*LMV* = Logarithm of market value

*MB* = Market to Book value

*Hightech* = 1 if the firm is in one of the high technology industries such as pharmaceuticals, aircraft and spacecraft, medical, precision and optical instruments, radio, television and communication equipment, office, accounting and computing machinery, and 0 otherwise.

*Lag_loss* = 1 when a firm's quarterly earnings report preceding the forecast is negative, and 0 otherwise

Taking a page out of Rakow [24], we converted *LMV* and *MB* as indicator variables that are set to one, if the value of the original variable is greater than or equal to the sample median, or zero otherwise.

Using dummy variables instead of continuous variables allows *α_1_* in equation (1) to be interpreted as the effect of the independent variable when the dummy variable is equal to zero, while *α_4_* through *α_7_* can be interpreted as the effect of each variable when the dummy variable is equal to one.

#### The Relationship of Analysts' Recommendations and Frequency of Forecast Errors (H2)

H2 is tested by running the following logit regression:
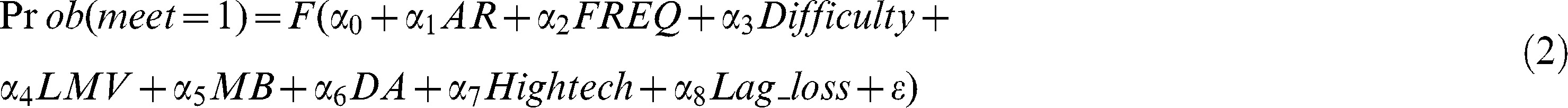
Where,

FEs are represented by the variable *meet*, which equals 1 if a firm's actual earnings meet or exceeds the management's forecasts, and 0 if otherwise.

*DA* is the firm's ability to manipulate earnings, as reflected by its discretionary accruals, which makes it ideal as a control variable. We use a version of the cross-sectional modified Jones model which is introduced by Ye [25] for the purpose of estimating discretionary accruals.

Other variables are similar to what was explained for equation (1).

#### The Relationship of forecast management and forecast errors (H3 and H4)

For the purpose of testing H3, the ANOVA will be used to test the difference of the mean value of FM between the companies that meet or miss forecasts in sell companies.

We will do the same test for buy companies in order to test the fourth hypothesis.

### Variables

There are three types of variables, such as independent, dependent and control variables that are being investigated in this study. Their respective measurements are discussed in this section.

#### Analysts' Recommendations (Independent Variable)

Following Heidle and Li [26], and Abarbanell and Lehavy [1], it is believed that the perception of the companies' future growth are duly reflected in the analysts' recommendations. Since analysts' recommendations fluctuates at levels less than the bid and ask spread [27] and ask and bid prices [26], it would remain unaffected by market sentiments, and it is assumed that it would be more reliable in capturing the company's growth perspective.

Analysts' recommendations come in five forms, namely strong buy, buy, hold, sell and strong sell. The rating assigned to each recommendation is displayed in Table 1.

**Table 1:** Recommendations and their assigned ratings.

Recommendations	Strong buy	buy	hold	Sell	Strong sell
**Rating**	1	2	3	4	5

Following Abarbanell and Lehavy [1], this research uses outstanding average (consensus) recommendations at the end of each day in the first, middle and last three weeks of the first month of the fourth quarter. The average recommendation for firm *i*, on date *t* is assumed to be *A_it_*.

Following Abarbanell and Lehavy [1], each observation is placed in one of the three categories. The first category consists of firms where *A_it_≤2* (denoted “Buy” stocks), the second category includes firms where *2<A_it_≤3* (“Hold” stocks), while the third contains the least favorably recommended firms, where *A_it_>3* (“Sell” stocks).

The number of buy and sell companies and the criteria for dividing them are shown in Table 2. In order to compare means (ANOVA tables) in section 5.3, since the extreme growth (buy) and non-growth (sell) companies are taken into account, the hold companies are omitted.

**Table 2:** The number of company years with buy, hold and sell recommendations.

	BUY	HOLD	SELL
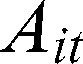 **Consensus analysts’ recommendations = **	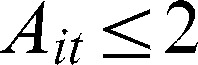	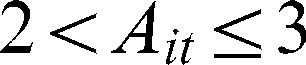	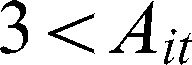
**No of companies in each category**	2078	3063	6278

The company years are divided to buy, hold and sell categories on the basis of consensus analysts’ recommendations.

#### Forecast Management (H1) (Dependent Variable)

Following Burgstahler and Eames [7] and Matsumoto [28], the proxy for FM is measured as follows:
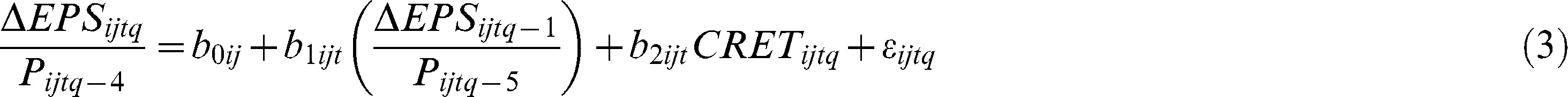
Where,

Subscripts refer to firm *i*, industry code *j*, quarter *q*, and year *t*, and

*ΔEPS_ijtq_* = earnings per share changes between the current quarter and four quarters prior.

*P_ijtq_* = price per share of common equity, and

*CRET_ijtq_* = cumulative daily excess returns from three days after the four quarters prior earnings announcement to 20 days before the current quarter earnings announcement.

*b_1ijt_* and *b_2ijt_* = the coefficients of the regression.

Similar to Matsumoto [28], (1) the model for each firm-year is estimated using all of the firm quarters of the year from the same industry, except those firms for which the parameters are estimated; (2) only firm-years with 10 or more firm-quarters of data in the same industry are included in the estimation, and (3) observations with variable values in the top and bottom half per cent of the respective distributions are omitted in order to mitigate the impact of extreme values on the parameter estimates. Then, the obtained parameter estimates were used to determine the expected earnings changes from the prior firm year's fourth quarter:
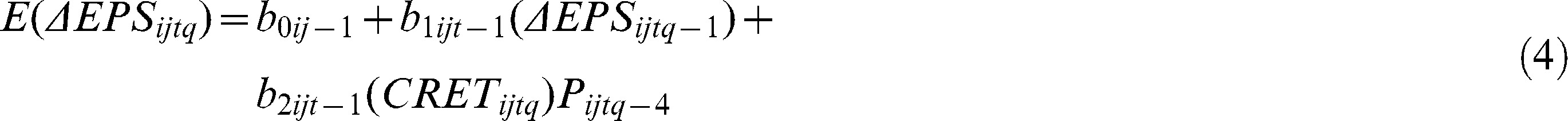



This expected change is added to earnings per share from the same quarter in the prior year in order to obtain the expected forecast of the current quarter's earnings:
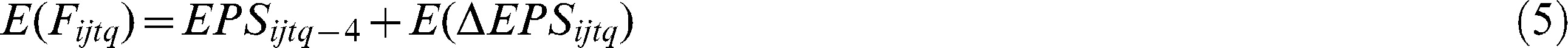



Consequently, in order to obtain the expected forecast of annual earnings, we estimated the fourth quarter expected earnings (from equation (5)), and added the prior three quarters of earnings realizations. We took into account the differences between the last reported forecast, and the model-derived expected forecast as a proxy for FM. In order to avoid including preannouncements, forecasts that has been reported near the end of the year have been excluded from the sample.
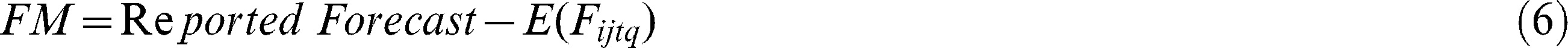



#### Forecast Errors (H2) (Dependent Variable)

According to Fang [29], Rogers and Stocken [30] and Xu [31], FE is calculated using the following formula:
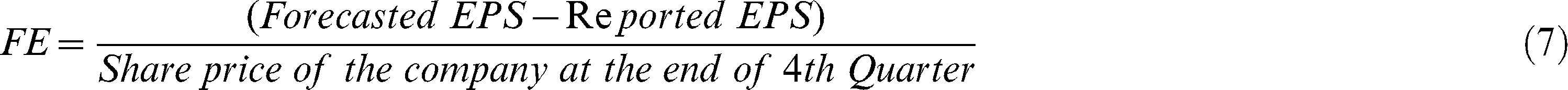
Forecasted EPS is the last forecast of the EPS that is reported by the management to the market.

#### Learning Effect (Moderating - Control Variable)

The market may learn from a firm's FM behavior over a period of time [30]. If the market discerns from a firm's history that it has habitually engaged in downward FM, they may expect the firms engaging in downward FM in its history to repeat this behavior, and carry out more downward FM than the cleaner firms. Consequently, market expectations will be weakly affected by the current FM. Thus, a rational manager may find it to be in their interest not to frequently manage forecasts downward [30], [32].

Therefore, we use a moderating variable, which reflects the frequency of forecast management (FREQ). Depending on the number of times the firm has conducted downward FM in the four previous periods, this variable can have the value of 1, 2, 3, or 4.

#### Difficulty (Moderating -Control Variable)

Difficulty reflects the degree of a market participants' ability to assess the credibility of the managements' forecast. According to Rogers and Stocken [30], factor analysis was used on several variables (indicators) for the purpose of identifying the difficulty construct. It is assumed that the indicator specific variances are uncorrelated across variables. Consistent with the goal of predicting FM, all variables are measured prior to the release of the forecasts. The following indicator variables generate a measure of forecasting difficulty [30]:

The standard deviation of analyst forecasts outstanding when the management forecast is released, *STD_AF*, measures the lack of analysts' consensus. The standard deviation of the previous analysts' forecasts errors, scaled by price for five years prior to the forecast release, *STD_AFE*, proxies for the difficulties analysts experienced when predicting earnings. It is more difficult to forecast a firm's earnings when the firm is unprofitable compared to when it is profitable. In order to recognize this asymmetry, the indicator *Lag-Loss* equals to 1 when a firm's quarterly earnings report preceding the forecast is negative, and 0 if otherwise. Also, the indicator of Predict-Loss equals 1 when the management forecast of earnings is negative, and 0 if vice versa. The standard deviation of the daily stock price for 120 days before the forecast date was measured and denoted as *STD_RET*. A firm's bid-ask spread is expected to increase with uncertainty regarding the firm's forthcoming earnings announcement (see [33]).

Table 3 reports the results of the factor analysis, when the continuous indicators of forecast difficulty are winsorized at 1 and 99 percent levels.

**Table 3:** Correlation Matrices and Factor Loadings for Forecast Difficulty Measure.

Panel A: Correlation Matrix for Forecast Difficulty Indicators						
	STD-AF	STD-AFE	Lag_Loss	Predict-Loss	STD-Ret	Spread
**STD-AF**		0.001	0.033*	0.052*	0.330**	0.001
**STD-AFE**	−0.001		−0.031	−0.075	0.970**	0.320**
**Lag-loss**	−0.014	0.144**		0.104**	−0.036	0.051*
**Predict-loss**	0.077**	0.050	0.104**		−0.054	−0.050
**STD-Ret**	0.330**	0.954**	0.171**	−0.029		0.954**
**Spread**	0.019	0.740**	0.188**	0.160**	0.748**	

Panel B: Factor Loadings						
Indicator	STD-AF	STD-AFE	Lagged-loss	Predict-loss	STD-Ret	Spread
**Factor Loading**	0.065	0.997	0.015	−0.021	0.997	0.118
**Standardized Factor Score**	0.039	0.958	0.112	−0.39	0.954	0.854

Panel C: Test of appropriateness of factor analysis					
**Total Variance Explained**	68.30%		**Bartlett's Test of Sphericity**	**Chi-Square**	50690
**Kaiser-Meyer-Olkin Measure of Sampling Adequacy**	.620			**Sig.**	0.000**

*, ** Significant at 5% and 1% level.

All of the significant correlations among the indicators possess their expected signs. The Difficulty latent variable is estimated by using Principal Axis Factoring (PAF). Standard factor analysis heuristics (e.g., scree-plots and eigenvalues) suggested three factors, and after accounting for the sign and magnitude of the factor loadings, the first factor is extracted as a measure of forecast difficulty.

The values for difficulty ranges from −0.58 to 0.84, where the lower value for this variable represent less difficulty for market participants to assess the credibility of management's forecasts, while higher values are indicative of higher level of difficulties.

#### Other Control Variables

One of the other control variable is the threat of litigation. Soffer et al.[34] stated that firms in a litigious environment want to prevent a large disappointment in the earnings announcement date, and this might be better accomplished by providing a less optimistic or even pessimistic forecast shortly before the earnings release date.

Kasznik and Lev [35] posits that firms in high-tech industries face higher risk of litigation as they experience larger price fluctuations, which might translate into potential losses to the investors. Similarly, Baginski et al. [36] used the high-tech industries to control the potential firm-specific litigation risks. The earnings of high-tech firms are more volatile, and inherently carry greater risks of inaccurate forecasts; all these factors could affect a firm's cost of capital. Therefore, a negative coefficient is predicted vis-à-vis high-tech, implying that high technology firms issue less optimistic forecasts.

In addition, similar to Rogers and Stocken [28], market to book value (MB), and loss in the previous period (Lag_loss) are used as control variables.

### Data and sample

The company's stock trading information, along with the forecast data, is gathered from the Bloomberg database. The potential market that was considered for data collection is companies in the New York Stock Exchange (NYSE).

The Bloomberg database is used to identify 14414 annual financial statements that were released between January 2005 and December 2010. From this number of companies, the AR for 11419 companies were made available (table 2).

Since firms in regulated industries are more likely to have different incentives than non-regulated industries [28], regulated industries, including utilities, transportation companies, and financial services are excluded from the sample [37], [38]. Companies with insufficient data in Bloomberg database are also excluded.

Among the remaining company-years, the Bloomberg database was searched for management earnings estimates, and actual (realized) earnings. The database was also searched for data regarding the analysts' recommendations, along with other relevant financial data pertaining to this work.

Based on the availability of the aforementioned data, and to carry out prediction tests, which involves examining forecasts reactions to analysts' recommendations, a subsample of 6553 forecasts were used. The sample selection procedure is summarized in Table 4.

**Table 4:** Sampling procedure.

**Number of all company-years in NYSE (2005–10)**		14414
**Less:** Companies that their AR are not available		(2995)
Number of the companies for which AR is available (Table 2)		11419
**Less:** Utilities, transportation or financial service	2238	
Forecasts are not available	215	
Forecasts issued less than one month prior to the end of fiscal year	823	
Insufficient data to calculate standard deviation of analysts’ forecasts	488	
Missing data for control variables on Bloomberg	265	
Insufficient time-series data on Bloomberg	603	
Forecasts that are not in quarter 4	234	
		(4866)
**Sample company-years for testing hypotheses**		6553

The hypotheses were tested in two subsamples. The first subsample was 6553 company-years, while the second subsample was 2449 company-years, which were in the vicinity of zero forecast errors. The reason for using the second subsample is explained in section 5.

## Findings

Burgstahler and Eames [36] argued that the benefit of FM to a firm may increase the amount of FM, i.e. there may be incremental benefits to beating rather than just meeting the analysts' forecasts. However, FM also imposes a cost on the firm. If there is a sudden drop in the marginal benefit at the point just to the right of the zero surprise point for many firms, then zero surprise is the optimal level that a firm should realize by conducting FM. Realistically, this scenario is entirely possible. The benefit(s) for the firms to just meet expectations is much larger than that for the firms just failing to meet expectations by a small margin, whereas the benefit to firms that beat expectations is only marginally larger than that of the firms that just barely meet expectations. This argument implies that firms that just meet expectations are more likely to have conducted FM, compared to firms that just fail to meet expectations and firms that do beat expectations.

Therefore, the main statistical tests are divided into two parts. In the first part, the hypotheses are tested by taking into account all of the involved company-years (first subsample). In the second part, the hypotheses are tested by considering the company-years that are in the vicinity of zero forecast errors (second subsample).

Since the distance near zero forecast error should be very small, and also since enough number of companies should be considered for analysis, the distance of 0.5 standard deviation of forecast error on the left and right side of zero forecast error is taken as small distance around zero FE.

### Relationship of analysts' recommendations and forecast management (H1)

Table 5 reports the results for the logistic regression analysis of FM (equation 6).

**Table 5:** Results for the Management Forecast Bias Hypothesis (Hypothesis 1).

Model	*Prob(Down = 1) = F(α_0_+α_1_ AR+α_2_AR×Difficulty+α_3_AR×FREQ+α_4_ LMV+α_5_ MB+α_6_ Hightech+α_7_ Lag_Loss+ε)*							
Dependent Variable: *(Down = 1)* if forecast management is negative and *(Down = 0)* otherwise								
Variable	Predicted sign	Coefficients			p-values		Marginal effects	
		*First Subsample*	*Second Subsample*		*First Subsample*	*Second Subsample*	*First Subsample*	*Second Subsample*
**Independents**								
*Constant*	?	1.034	−1.75		0.276	0.892	−	−
*AR*	+	0.689	0.699		0.01**	0.000***	1.993	2.012
**Control Variables**								
*AR* * *Difficulty*	+	3.25	2.662		0. 03***	0.021**	2.577	1.282
*AR* * *FREQ*	−	−0.115	−0.151		0.114	0.019**	0.891	0.86
*LMV*	+	0.155	0.353		0.422	0.023**	1.167	1.424
*M/B*	−	−0.006	−0.003		0.654	0.686	0.994	0.995
*Hightech*	+	−0.18	−0.181		0.399	0.102	0.835	0.834
*Lag_Loss*	+	0.499	0.739		0.044**	0.098*	1.647	1.538
Log Likelihood		564.693	564.435		**Hosmer Lemeshow**			
Chi-square		57.602	59.383	Pearson χ^2^	510.36	510.13		
P-value		0.000***	0.000***	Prob	0.243	0.246		

Logistic regression results of the first and second subsamples. The coefficients and related t-statistics are estimated by using the following model: *Prob(Down = 1) = F(α_0_+α_1_ AR+α_2_AR×Difficulty+α_3_AR×FREQ+α_4_ LMV+α_5_ MB+α_6_ Hightech+α_7_ Lag_Loss+ε).*

*, **,*** Significant at 0.1, 0.05 and 0.01 levels, respectively based on one-tailed tests for signed predictions, two-tailed tests otherwise.

The interaction term *AR* and *FREQ* is used to measure the effect of learning from historical FM on the relationship between *AR* and FM. Thus, the algebraic expression for equation (5) is that *α_1_* is positive. However, the algebraic expression of learning effect is that α_3_ is negative and significant. Within this specification, the coefficient of FM to *AR* should be *α_1_+α_3_×FREQ*.

The coefficient of FM to *AR* for a non-difficult firm is *α_1_*. However, the coefficient of FM to *AR* for a difficult firm is *α_1_+α_2_*.

Taking into account Table 5 for the first subsample, the coefficient of *AR* is positive and significant at a 5% level, for the second subsample, the coefficient of *AR* are positive and significant at a 1% level. This implies that when the *AR* for the company is high (i.e. the company is in sell position), the companies conduct higher income decreasing FM compared to when the *AR* is low (the company is in buy position). The coefficients of *AR×Difficulty* are significantly positive in both subsamples (subsample of all company-years and subsample of company-years, which are near zero FE), implying that when it is more difficult for analysts and investors to forecast the company's profit, and thus recognize the credibility of the management forecasts, the management will do more income decreasing FM. Additionally, the coefficient of frequency (*AR×FREQ*) is significant and negative in the second subsample. This shows that the frequency of the previous year's FM moderates the relationship between *AR* and FM.

Thus, as a result of the significance of the coefficients of *AR* in both subsamples, it is concluded that *AR* affects FM, and the first hypothesis is supported. In addition, difficulty and frequency moderate the relationship between *AR* and FM in companies in a small distance around zero FE. This result shows that managers strategically manipulate their forecasts downward, making it more difficult for the market to assess the truthfulness of their disclosure.

With respect to control variables, the coefficients of *lag_Loss* are significantly positive for the first subsample at 0.05 and for the second subsample at 0.1. This means that the companies that experience lagged loss conduct more downward FM compared to other companies. Also, in the case of companies in a small distance around zero FE, the coefficient of *LMV* is significant at a 0.1 significance level, and possess its expected values. The coefficients on the remaining control variables are rather insignificant.

The marginal effects are analogous to the slope's coefficients in an OLS regression [35]. The marginal effect for AR is 1.993 and 2.012 for first and second subsamples, respectively, suggesting that moving from the first to the third quartile of *AR*, the probability of meeting or exceeding expectations increases by approximately 99 and 101 percent, respectively. The values of the marginal effects of *AR×Difficulty* are 2.577 and 1.282 for the first and second subsamples, respectively. They indicated that the companies that the credibility of their management's forecasts are most difficult to be assessed by market participants, the probability of its FM is approximately 2.57 and 1.28 times more than the least difficult firms.

The Hosmer and Lemeshow test was used to test the fitness of the models. The test result shown in the lower part of Table 5 is not significant for any of the models, confirming the goodness of fit of the models. Additionally, in order to see the robustness of the results, after dropping each one of the control variables, there were no significant changes in the robustness of the model.

In addition, for determining the presence of multicollinearity, the VIF statistics for independent variables in regression (1) (untabulated) demonstrated no sign of high correlation between independent and control variables.

### Relationship of analyst's recommendations and frequency of positive forecast errors (H2)

To examine the relationship of analysts' recommendations and the frequency of positive FEs, analogous to Matsumoto [26], and by using a cross-sectional logit regression, the regression in equation (2) is estimated (firm and time subscripts have been suppressed):

The results of the logit regressions are indicated in Table 6.

**Table 6:** Logit analysis of the probability of meeting or exceeding forecasts and the incentives to avoid negative FEs.

Model	*Prob(meet = 1) = F(α_0_+α_1_ AR+α_2_ FREQ+α_3_ Difficulty+α_4_ LMV+α_5_ MB+α_6_DA+α_7_Hightech+α_8_ Lag_loss+ε)*							
Dependent Variable: *(meet = 1)* if the reported profit meets of exceed forecasts and zero otherwise.								
Variable	Predicted sign	Coefficients			p-values		Marginal effects	
		*First Subsample*	*Second Subsample*		*First Subsample*	*Second Subsample*	*First Subsample*	*Second Subsample*
*Intercept*	?	−1.694	−1.217		0.046	0.198	−	−
**Independent**								
*AR*	+	0.374	−0.381		0.136	0.072*	1.235	0.464
**Control Variables**								
*FREQ*	−	−0.981	−0.890		0. 002***	0.002**	0.375	0.411
*Difficulty*	+	0.832	0.960		0.001***	0.001**	1.879	2.117
*LMV*	+	0.147	0.200		0.344	0.355	1.159	1.221
*MB*	+	0.004	0.003		0.596	0.540	1.004	1.003
*DA*	+	0.000	0.021		0.590	0.816	1.000	0.899
*High-tech*	−	0.345	0.288		0.121	0.117	1.412	1.334
*Lag_loss*	+	0.555	0.693		0.077*	0.028*	0.742	0.999
*Year*	+	0.571	0.561		0.015	0.016*	1.771	1.762
Log Likelihood		544.183	544.018		**Hosmer Lemeshow**			
Chi-square		170.837	171.032	Pearson χ^2^	4726.44	639.78		
P-value		0	0.000	Prob	0.3028	0.6263		
Meet/Exceed		3043	1372					
Did not meet		2623	1077					

The regression is run on the first and second subsamples. The coefficients and related t-statistics are estimated by using the following model:

*Prob(meet = 1) = F(α_0_+α_1_ AR+α_2_ FREQ+α_3_ Difficulty+α_4_ LMV+α_5_ MB+α_6_DA+α_7_Hightech+α_8_ Lag_loss+ε).*

*, **,*** Significant at 0.1, 0.05 and 0.01 levels, respectively based on one-tailed tests for signed predictions, two-tailed tests otherwise.

In Table 6, the coefficient on *AR* is positive but non-significant for the first subsample, however, for the second subsample, the coefficient of *AR* is negative and significant suggesting that buy companies are more likely to meet forecasts. Contradicting expectations, in small distance around zero FE, sell companies do not possess higher positive forecasts errors. Thus, the second hypothesis is not supported. The reason might be that buy companies might have used income increasing earnings management strategy to meet the forecasts. In order to produce positive forecasts errors, the income increasing earnings management in buy companies might have been more efficient than income decreasing FMs in sell companies. The coefficient of *FREQ* is negative and significant at 1% in both subsamples, indicating that if a company has high frequency of income decreasing FM in the previous years, the probability of having positive FE decreases in the current year. This confirms Rogers and Stocken [30] findings that managers have fewer incentives to avoid negative surprises when the frequency of downward FM in previous years is high. Additionally, for both subsamples, the coefficient of Difficulty is positive and significant, which is consistent with Rogers and Stocken [30] notion that managers have more incentives to conduct FM, and thus avoid negative surprises when the recognition of FM is more difficult for investors. The coefficient of *lag_loss* is negative and significant, consistent with the conjecture that those firms with low value-relevance of earnings have less incentive to avoid negative FEs. The positive but insignificant coefficient of *Hightech* implies that firms with relatively higher litigation prospects appear to be marginally more likely to avoid negative FEs.

Columns 7 and 8 report the marginal effect of each variable. It is analogous to the slope coefficients in an OLS regression [35]. The marginal effects for frequency are 0.375 and 0.411. These values suggest that moving from the first to the third quartile of *FREQ* decreases the probability of meeting or exceeding analysts' expectations by approximately 62.5 and 58.9 percent in the first and second subsamples, respectively. The marginal effect for difficulty equals 1.879 and 2.117 in the first and second subsamples, indicating that an increase in the difficulty of predicting future profits increasing the probability of meeting or exceeding analysts' expectations by 87 and 111 percent, respectively. The marginal effect for *Lag_loss* equals to 0.742 and 0.999, implying that in firms that reported losses in the previous period, the probability of meeting or exceeding analysts' expectations is lower by 26 and 1 percent in the first and second subsamples, respectively.

### Relationship of forecast management and forecast errors in buy and sell companies (H3, H4)

For testing the third hypothesis, the difference of mean values of FM of the companies that possess zero or positive FEs (meet or beat forecasts), and the companies that possess negative FEs (miss forecasts) in sell groups are tested. Columns 3 to 5 of Table 7 show the results of the test.

**Table 7:** Test of difference in mean forecast management for the companies that meet management forecasts and the companies that miss forecasts in the Sell (H3) and Buy (H4) companies.

			Sell			Buy		
			No.	Mean	STDEV	No.	Mean	STDEV
**Positive or zero FE**	***FM***	**First Subsample**	2764	−0.0067	0.105	936	−0.0012	0.0768
	***FM***	**Second Subsample**	908	−7.09E-03	0.0080	394	5.59E-03	0.00658
**Negative FE**	***FM***	**First Subsample**	2593	0.0032	0.15	828	0.0065	0.1128
	***FM***	**Second Subsample**	732	7.68E-03	0.0135	298	2.46E-02	0.01338
				**ANOVA’s F**	**Sig.**		**ANOVA’s F**	**Sig.**
	***FM***	**First Subsample**		0.098	0.756		1.222	0.274
	***FM***	**Second Subsample**		5.1830	0.023**		2.7457	0.098*

*, ** : Significance at 0.1 and 0.05.

Tests of differences in mean values of FM between companies that meet or beat forecasts, and the companies that miss forecasts in the group of sell companies showed that there is no significant difference in the mean values of FM between them for the first subsample. However, in the second subsample, the companies that meet forecasts have significantly lower value of FM compared to companies that misses forecasts. This means that in sell companies of subsample 2, the companies that meet or beat forecasts possess more downward FM than companies that fail to meet their forecasts. Thus, in this subsample, H3 is supported.

In addition, columns 6 to 8 of Table 7 shows the result of testing the difference in mean values of FM between the companies that possess zero or positive FEs, and the companies that possess negative FEs in the buy group.

Tests of difference in the mean values of FM between companies that meet or beat forecasts, and the companies that miss their forecasts in the group of buy companies showed that there is no significant difference in the mean value of FM between them for the first subsample. For the second subsample, although the difference is significant at a 10% significance level, it is not strong enough to reject the fourth hypothesis. Therefore, H4 is supported.

## Discussion

The findings of this research shows that income decreasing FM is more evident in sell firms (H1). Sell companies conduct income decreasing FM to avoid negative forecasts errors and its consequent market punishments that unfavorably affects the management's utility (H3).

However, avoiding negative forecasts errors via conducting income decreasing FM is not evident in buy firms (H4). This result is consistent with Abarbanell and Lehavy [1], who found that instead of carrying out income decreasing FM, buy companies conduct income increasing earnings management to meet forecasts and produce positive FEs.

### Behavior of the buy companies

The findings for (H1) indicate that in buy companies, management conveys less pessimistic forecasts to the market. In addition, the findings for (H4) showed that there is not a significant difference in FM between the buy companies that meet or miss their forecasts. Since for buy companies that have favorable financial records, investors are more responsive to forecasted news, such firms would like to have their private information more fully impounded into their stock prices, and consequently are more capable of reducing information asymmetries in the market, and enjoy lower cost of capital [33], [39], [40].

Assuming that the management seeks to align market expectations with their own (see Ajinkya and Gift [41]), it is especially true that when the management have extremely promising news to convey [42], and therefore, a favorable track record will be most helpful in enhancing the forecasts' credibility of buy companies. The reason might be due to the fact that by conveying true information regarding their favorable records, buy companies' private information, which is usually promising, is fully impounded into their stock prices, and consequently, they are more capable of reducing information asymmetry and enjoy lower costs of capital. Additionally, buy companies are able to do income increasing earnings management to meet the forecasts. Therefore, they need to do less income decreasing FM than sell companies. This might be interpreted as the discovery of the fact that buy companies convey a less pessimistic forecast to the market.

### Behavior of the sell companies

The sell companies conduct high income decreasing FM (H1 supported) to realize positive forecasts errors (H3 supported). The reason might be that sell companies issue pessimistic forecasts to avoid the unfavorable utility minimizing consequences of missing forecasts.

In other words, sell companies conduct downward FM to avoid market punishments that results from missing forecasts [16], [43]. The reason for this is that as mentioned in section 1 (Introduction), sell companies do not usually generate high economic profits. Therefore, the pessimistic forecasts of sell companies are to avoid market punishments, rather than being opportunistic.

According to Dutta and Gigler [2] framework, the pessimistic forecasts of sell companies might not be due to opportunism. Such pessimism makes their reporting process to be consistent with the efficiency perspective that corresponds with the revelation principle. Therefore, consistent with Dutta and Giggler's [2] proposition, it is optimal to render income increasing earnings management potentially costly for sell (non-growth) companies, so that they do not report delusive optimistic forecasts.

## Conclusion

This research adds to the literature by finding an additional factor that affects management decisions toward issuing forecasts. It has been found that the companies' growth statuses that are represented by analysts' recommendations (as buy or sell recommendations) can affect the managements' decision to conduct FM.

This study helps to understand the mixed findings in the management forecasts literature. While the previous studies suggested that management forecasts are opportunistic, and the management uses the forecasts to manage the analysts' forecasts [44], [45] and affect the stock prices [46], there are several other studies that showed that since management's forecast conveys insider information to the outsiders, it helps to lessen information asymmetry, hence decreasing costly litigation of the stockholders against the company [16], [43]. It also helps the company to have clear and transparent financial reporting [3], [12]. This study adds to the mixed findings in the literature by demonstrating that the management's forecasts contains a bias that is predictable, taking into account the analysts' recommendations about the company.

This research is practically useful, as it extends the existing knowledge regarding the information content of the management's forecasts that affect decisions of the users of the financial information. The findings warn investors to carefully evaluate the management's forecasts on the basis of whether the companies have buy and sell recommendations before they form their expectations about the company. The findings suggest that the information regarding the analysts' recommendations might contain important implications for FM, as they might convey informational values that can be used by researchers or even investors.

While the present study reveals some significant points in terms of reliability and accuracy of management forecasts, the findings should neither be overestimated nor underestimated. Gathering data from different markets and from different time periods, and using different FM measurement models might illuminate the issue of the reliability of management forecasts.
